# Case Report: Unraveling a web of clots: marantic endocarditis as a paraneoplastic manifestation of lung cancer

**DOI:** 10.3389/fonc.2025.1755655

**Published:** 2026-01-12

**Authors:** Bart Forier, Frederik Staels, Dorian Bivort, Mike Ralki

**Affiliations:** Department of Pneumology, AZ Sint-Maria Hospital, Halle, Belgium

**Keywords:** EGFR, endocarditis, hypercoagulability, lung cancer, non-bacterial thrombotic endocarditis (NBTE)

## Abstract

Marantic or non-bacterial thrombotic endocarditis (NBTE) is an uncommon but clinically important complication of malignancy, driven by a profound hypercoagulable state. It occurs most frequently in advanced adenocarcinomas and can present with a wide spectrum of thrombotic events, often mimicking infective endocarditis or other embolic disorders. We describe a rare presentation of metastatic EGFR-mutated lung adenocarcinoma complicated by NBTE, pulmonary embolism, splenic infarction, and acute coronary artery thrombosis. The diagnosis was supported by negative blood cultures, evidence of systemic emboli, and echocardiographic detection of a tricuspid valve vegetation. Management included prompt anticoagulation and initiation of targeted EGFR-directed therapy, which resulted in significant clinical and radiologic improvement, including complete resolution of the valvular lesion. This case underscores the need for heightened clinical suspicion for NBTE in patients with malignancy or unexplained embolic events, particularly when sterile valvular vegetations are identified. It also highlights the central role of effective cancer treatment—alongside anticoagulation—in reversing the prothrombotic state that drives NBTE.

## Introduction

Non-bacterial thrombotic endocarditis (NBTE) or marantic endocarditis is a rare condition characterized by sterile valvular vegetations that predispose to systemic embolization. It is most commonly associated with advanced malignancy, particularly adenocarcinomas of the lung, pancreas, and gastrointestinal tract (1–4). Tumor-related procoagulants and a hypercoagulable state drive vegetation formation and thrombotic complications (5).

NBTE is challenging to diagnose because symptoms often mimic infective endocarditis or thromboembolic disease. Echocardiography, negative blood cultures, and evidence of systemic emboli are essential for diagnosis (1, 6, 7). Early recognition is critical, as anticoagulation and treatment of the underlying malignancy can improve outcomes (1, 6, 7).

## Case description

A previously healthy 39-year old woman presented to the emergency department with acute right-sided pleuritic chest pain and progressive exertional dyspnea (mMRC grade 3). Additionally, she reported mild epistaxis, right arm weakness, and blurred vision. She had recently returned from a one-month stay with her father in Sydney, Australia, during a period of intense bushfires. Prior to travel, she had experienced rhinitis, sinusitis, and minor hemoptysis. Shortly after arrival in Australia, she developed pleuritic pain on the right side of the chest. Empirical treatment with azithromycin and subsequently with amoxicillin for a right-sided pulmonary consolidation on chest radiograph yielded no improvement.

On admission, her blood pressure was 169/87 mmHg, heart rate 87 bpm, oxygen saturation 99% on room air, and temperature 36.7°C. Cardiopulmonary examination was unremarkable, without edema or signs of deep venous thrombosis. A faint erythematous rash was noted on the neck. Laboratory testing revealed a markedly elevated D-dimer level (25,585 ng/mL, ref value < 500 ng/mL). and a mildly increased CRP (34 mg/L, ref value <5 mg/L).

Given the combination of dyspnea, pleuritic pain, a recent long-haul flight and high D-dimer, pulmonary embolism (PE) was suspected. Differential diagnoses included complicated pneumonia, vasculitis, systemic lupus erythematosus, and malignancy.

**Figure 1:**
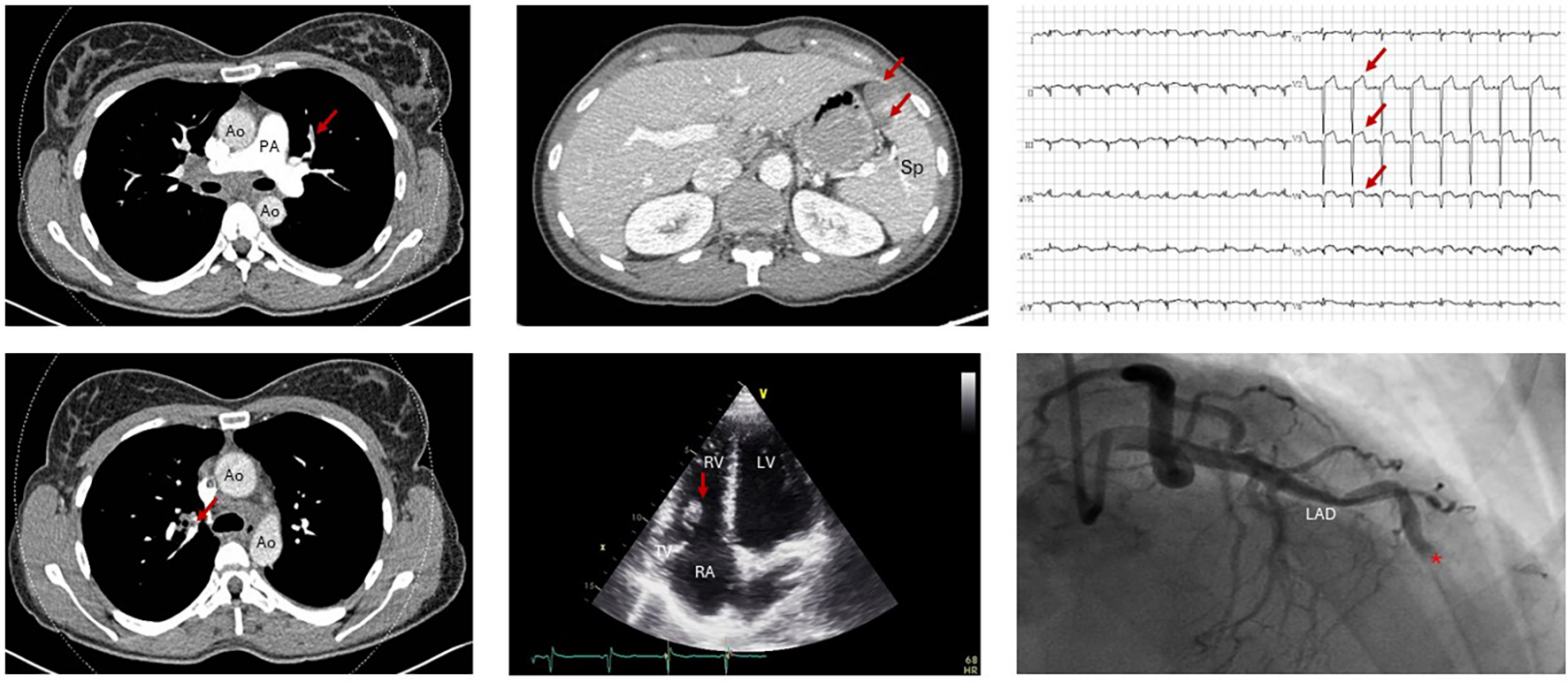
Thrombotic complications. Left: Contrast-enhanced chest CT showing bilateral segmental pulmonary embolism (red arrows). PA, pulmonary artery; Ao, aorta. Middle: Top: Contrast-enhanced abdomen CT showing multiple splenic infarcts (red arrows) and (not shown) hepatic infarcts. Sp: spleen. Bottom: TTE showing presence of a mobile, rounded vegetation (11x16mm) on the subvalvular apparatus of the tricuspid valve at the antero-posterior leaflets (red arrow). RA, right atrium RV, right ventricle LV, left ventricle TV, tricuspid valve. Right, Top: ECG showing ST-elevation myocardial ischaemia in anteroseptal leads V2,3 and 4 (red arrows). Bottom: Coronary artery catheterisation with right anterior oblique view shows total occlusion of left anterior descending artery (LAD) (red asterix).

CT pulmonary angiography demonstrated bilateral pulmonary emboli, predominantly in the left lower lobe, with right-sided parenchymal consolidation and pleural effusion suggestive of pulmonary infarction (**Figure 1**). Anticoagulation with low-molecular-weight heparin (enoxaparin twice daily 1mg/kg) was initiated for low-risk PE. Transthoracic echocardiography (TTE) on day 6 revealed normal biventricular function and no signs of pulmonary hypertension (sPAP 28 mmHg) but identified a mobile mass attached on the subvalvular apparatus of the tricuspid valve at the antero-posterior leaflets, raising suspicion of thrombus or non-infective endocarditis (**Figure 1**). There was no evidence of valvular dysfunction. Blood cultures were negative and there were no clinical signs of infection.

**Figure 2:**
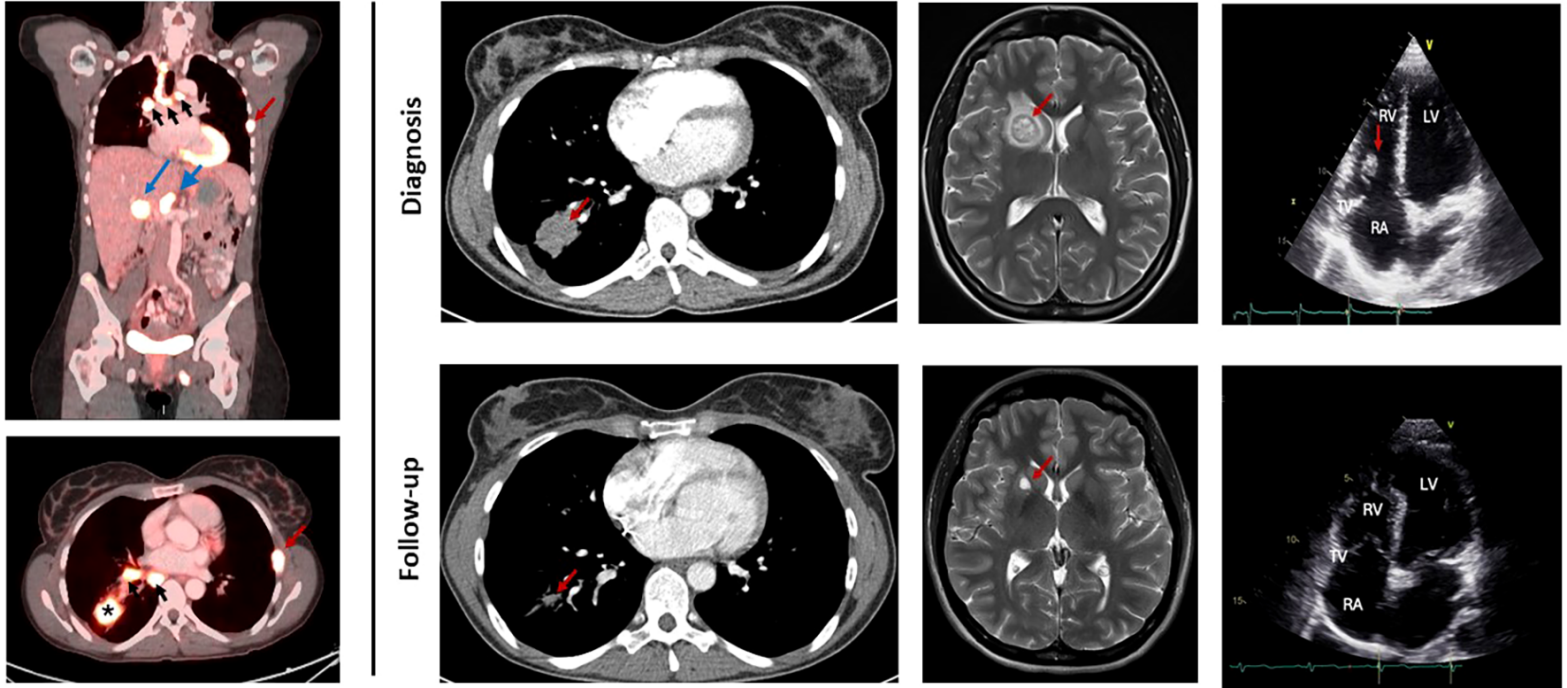
Oncological diagnosis and response to treatment. Left panel: PET-CT at diagnosis showing intense FDG-captation of the consolidation in the right lower lobe (black asterix), ipsilateral and contralateral mediastinal (black arrows) and hepatic hilar lymphadenopathies (blue short arrow), liver metastasis (blue long arrow) and multiple bone metastases (red arrow). Right panel: Treatment response contrast enhanced chest CT (left) showing volume reduction of primary tumor in the right lower lobe (red arrow) and brain MRI T2-weighted, contrast-enhanced image (middle) showing volume reduction of brain metastasis in the right lentiform nucleus (red arrow) after 1 year of treatment with Osimertinib. Transthoracic echocardiography, apical 4 chamber view (right) showing tricuspid valve vegetation at diagnosis (red arrow) and no residual vegetation after 4 months of treatment with Osimertinib. RA, right atrium. RV, right ventricle. LV, left ventricle. TV, tricuspid valve.

Subsequent PET-CT on day 13 showed extensive FDG-avid supraclavicular, axillary, mediastinal, and hilar lymphadenopathy, along with hypermetabolic hepatic and osseous metastases and a metabolically active right-sided pulmonary mass (**Figure 2**). Multiple right-sided pulmonary infarcts were FDG-negative. Additionally, splenic infarction was detected. Brain MRI revealed numerous small metastases and one larger lesion with perilesional edema causing mild mass effect on the right lateral ventricle (**Figure 2**). High-dose corticosteroid therapy (methylprednisolone 32 mg daily) was started for symptomatic cerebral edema on day 13.

Endobronchial ultrasound-guided transbronchial needle aspiration (EBUS-TBNA) from mediastinal lymph nodes (stations 7 and 4L) on day 18 confirmed lung adenocarcinoma on morphology and immunohistochemistry. Immunohistochemical staining showed high PD-L1 expression (PD-L1 TPS 90%), ALK and ROS1 negativity. Rapid EGFR testing (Idylla) demonstrated an EGFR exon 19 deletion on day 22, which was confirmed by next-generation sequencing on day 30.

On day 21, the patient developed acute anterior ST-segment elevation myocardial infarction. Coronary angiography (**Figure 1**) revealed complete occlusion of the left anterior descending artery due to thrombus without any underlying atherosclerotic disease. Thrombus aspiration was performed, and post-infarct echocardiography showed a reduced left ventricular ejection fraction of 30%. Guideline-directed heart failure therapy was initiated with low-dose bisoprolol (2.5 mg) and ramipril (2 × 1.25 mg), but titration was limited by hypotension.

Targeted therapy with osimertinib (Tagrisso) 80 mg once daily, a third-generation EGFR tyrosine kinase inhibitor, was initiated on day 22. The treatment was well tolerated, leading to progressive clinical improvement and discharge on day 33. At discharge, enoxaparin (Clexane) twice daily was transitioned to once-daily tinzaparin (Innohep) to facilitate outpatient treatment.

Follow-up imaging one month later demonstrated marked regression of mediastinal lymphadenopathy and shrinkage of the primary lung lesion, accompanied by a decrease in serum CEA from 294 µg/L to 145 µg/L (ref < 5 µg/L). Repeat TTE revealed significant reduction of the tricuspid valve mass (from 11×16 mm to 3×12 mm), consistent with marantic (non-bacterial thrombotic) endocarditis secondary to metastatic lung adenocarcinoma.

Subsequent follow-up demonstrated further gradual recovery. TTE (**Figure 2**) and cardiac MRI 4 months after initial presentation showed complete resolution of the previously observed tricuspid valve vegetation. At that stage, tinzaparin was transitioned to edoxaban (Lixiana 60 mg once daily) to provide long-term anticoagulation in the context of ongoing metastatic disease. Consecutive chest CT scans and brain MRI on 4, 6 and 12 months after presentation revealed persistent partial remission with marked reduction of the primary pulmonary tumor and regression of cerebral metastases (**Figure 2**). In the setting of persistent post-infarction systolic dysfunction with a left ventricular ejection fraction of 35%, an implantable cardioverter-defibrillator (ICD) was implanted for primary prevention of sudden cardiac death after multidisciplinary discussion, taking into consideration the patient’s favorable oncological response.

## Discussion

Non-bacterial thrombotic endocarditis (NBTE) represents a rare but clinically significant manifestation of cancer-associated coagulopathy. Prevalence estimates of NBTE vary from 0.3% to 9.3% in autopsy studies (8). In a recent large autopsy series of 50,403 patients, NBTE was identified in 3.7%, approaching the frequency of infective endocarditis, yet none were diagnosed ante-mortem (9). NBTE was strongly associated with malignancy (59% of cases, predominantly adenocarcinomas, 62%) and pre-existing valvular degeneration (9). NBTE is characterized by sterile, fibrin–platelet vegetations on cardiac valves that are prone to embolization, leading to systemic infarctions in multiple organs (1, 2, 10, 11). Although NBTE has been described in association with various malignancies, adenocarcinomas—particularly of the lung, pancreas, and gastrointestinal tract—account for the majority of cases (1, 2, 10, 12).

NBTE arises at the intersection of endothelial injury and a profound prothrombotic milieu. Tumor-derived procoagulant factors (including tissue factor and cancer procoagulant), inflammatory cytokines, and circulating mucins promote platelet and fibrin deposition on otherwise intact valve leaflets (5). Platelet–fibrin aggregates form sterile vegetations composed largely of fibrin and platelets with minimal inflammatory infiltrate; these vegetations are friable and prone to embolize. Clinical and translational studies have also linked specific tumor biology to thrombotic risk — for example, adenocarcinomas and certain oncogenic alterations (ALK, ROS1 rearrangements in lung cancer) may upregulate tissue factor expression, thereby increasing thrombotic propensity (13). The role of EGFR mutations, as seen in our case, in thrombotic risk, remains controversial. A large meta-analysis encompassing over 21,000 patients concluded that EGFR-mutant non-small cell lung cancer (NSCLC) is not significantly associated with increased venous thrombo-embolism (VTE) risk (14). In contrast, in one cohort, EGFR mutation emerged as an independent risk factor for postoperative VTE (10.2%) (15). Patients with NBTE commonly present with embolic phenomena (stroke, limb or visceral infarcts) or with symptoms arising from the underlying malignancy; constitutional features (eg. weight loss, night sweats) and minor pulmonary symptoms (as in the current case) are frequent antecedents. A recent study underscores the close association between NBTE and advanced lung cancer (16). In a review of 32 cases, 86% had stage IV disease at the time of NBTE diagnosis. Nearly half of the patients presented with stroke as the initial event, and over 60% had multi-organ infarctions, most frequently affecting the brain. The aortic and mitral valves were most often involved, while tricuspid valve lesions—such as in our patient—were uncommon (16).

Blood cultures are typically negative, fever is often absent, and cardiac auscultatory findings may be minimal — features that help distinguish NBTE from infective endocarditis (3, 6, 7). Our patient fulfilled the modified Duke criteria for possible infective endocarditis, with evidence of endocardial involvement and peripheral embolic phenomena. Nevertheless, the broader clinical picture — including an underlying malignancy, repeatedly negative blood cultures, and the progressive regression of the tricuspid vegetation following initiation of osimertinib — was consistent with NBTE. Transthoracic echocardiography (TTE) can detect valvular vegetations, but sensitivity is limited and small, mobile lesions are best seen on transesophageal studies (TEE) (3, 6, 7). In our case, TEE was not pursued as the tricuspid valve vegetation was already well visualized on TTE, with no signs of valvular dysfunction.

Therapy for NBTE rests on two pillars (1): prompt and effective anticoagulation to reduce further thromboembolisms, and (2) treatment of the underlying malignancy to correct the prothrombotic stimulus (1, 6). Historically, unfractionated heparin or low-molecular-weight heparin have been favored, both in older reports and in contemporary practice, because of (a) rapid onset, (b) evidence suggesting greater efficacy in cancer-associated thrombosis, and (c) greater experience in the setting of NBTE (17). Vitamin K antagonists are less favored, and the role of direct oral anticoagulants (DOACs) in NBTE is not yet well established — evidence remains limited and caution is advised, particularly for arterial embolic prevention in advanced cancer (17). When a targetable tumor driver is identified and effective systemic therapy (e.g., EGFR-TKI for EGFR-mutant lung adenocarcinoma in our case) can be initiated promptly, regression of the malignant process often accompanies reduction in thrombotic activity and sometimes leads to shrinkage or resolution of vegetations. Valve surgery is rarely indicated except for severe, refractory valvular dysfunction or recurrent embolism despite optimal anticoagulation and cancer therapy (1, 7, 18). In our case, surgical intervention was not pursued. This decision followed a multidisciplinary discussion including cardiology, cardiac surgery and oncology, during which the risks and expected benefit of operative management were weighed carefully. The patient’s malignancy-related hypercoagulable state implied a substantial likelihood of recurrence, particularly if prosthetic material were required, and cardiac surgery in this clinical context carried considerable peri-operative risk. In addition, the tricuspid valve lesion was clearly visualized on TTE, with no evidence of valvular dysfunction, and there was a realistic expectation of response to medical therapy with combined anticoagulation and prompt initiation of EGFR-targeted treatment. Given these considerations, a conservative strategy was preferred, aiming to control the underlying tumor biology while minimizing procedural risk.

Compared with previously reported cases of NBTE, this case is notable for several reasons. First, the diagnosis was made early in the clinical course, triggered by bilateral pulmonary emboli, an unusually high D-dimer, and a mobile tricuspid valve vegetation on TTE, whereas most published cases were diagnosed late or only at autopsy (19). Second, although our patient experienced systemic embolic complications similar to those reported in other cases, she did not develop ischemic stroke, which is the most frequently reported embolic manifestation of NBTE (19). In contrast, coronary thrombosis is rarely reported in NBTE, yet it was a significant complication in our patient, leading to persistent left ventricular dysfunction and the subsequent need for ICD implantation. A recent review of case series reported nine antemortem cases of myocardial infarction due to NBTE, approximately half of which occurred in patients with underlying malignancy, while the remainder were associated with autoimmune or other hypercoagulable conditions. Most patients presented with STEMI, and management strategies varied between percutaneous coronary intervention and conservative anticoagulation, depending on lesion accessibility and patient stability (20). Third, tricuspid NBTE in lung cancer is exceptionally rare, with only a few cases reported (21–23). In our case, the tricuspid vegetation was identified prior to the cancer diagnosis, and complete resolution was achieved with anticoagulation and targeted therapy alone, whereas previous cases either required procedural intervention or were recognized only at autopsy. Finally, reversible NBTE has rarely been described, typically in the context of effective cancer therapy. Three reported cases involved patients with EGFR-mutant or ROS1-rearranged lung adenocarcinoma, in whom NBTE vegetations regressed and coagulation parameters improved following molecularly targeted therapy combined with anticoagulation (24, 25). Our case similarly demonstrates complete resolution of tricuspid vegetation after initiation of EGFR-targeted therapy and anticoagulation, highlighting that early recognition and prompt oncologic treatment can reverse NBTE. Nevertheless, such reports are scarce, and the overall prognosis of NBTE remains poor, reflecting its strong association with advanced malignancy and high thromboembolic risk.

## Conclusion

This case report describes an exceptional presentation of EGFR-mutated metastatic lung adenocarcinoma complicated by marantic or NBTE, pulmonary embolism, splenic infarction and acute coronary artery thrombosis. The prompt initiation of targeted EGFR therapy combined with anticoagulation led to a radiologic and echocardiographic regression of disease and vegetations, illustrating the principle that control of the underlying malignancy is fundamental for the reduction of thrombotic complications.

## Data Availability

The original contributions presented in the study are included in the article/supplementary material. Further inquiries can be directed to the corresponding author.
